# Amyloid Cross-Seeding: Mechanism, Implication, and Inhibition

**DOI:** 10.3390/molecules27061776

**Published:** 2022-03-08

**Authors:** Sushma Subedi, Santanu Sasidharan, Niharika Nag, Prakash Saudagar, Timir Tripathi

**Affiliations:** 1 Molecular and Structural Biophysics Laboratory, Department of Biochemistry, North-Eastern Hill University, Shillong 793022, India; sushmasubedi22@gmail.com (S.S.); niharikanag.07@gmail.com (N.N.); 2 Department of Biotechnology, National Institute of Technology Warangal, Warangal 506004, India; santanu.sasidharan@gmail.com

**Keywords:** amyloid proteins, aggregation, cross-seeding, protein misfolding diseases, dual inhibition, fibrillation

## Abstract

Most neurodegenerative diseases such as Alzheimer’s disease, type 2 diabetes, Parkinson’s disease, etc. are caused by inclusions and plaques containing misfolded protein aggregates. These protein aggregates are essentially formed by the interactions of either the same (homologous) or different (heterologous) sequences. Several experimental pieces of evidence have revealed the presence of cross-seeding in amyloid proteins, which results in a multicomponent assembly; however, the molecular and structural details remain less explored. Here, we discuss the amyloid proteins and the cross-seeding phenomena in detail. Data suggest that targeting the common epitope of the interacting amyloid proteins may be a better therapeutic option than targeting only one species. We also examine the dual inhibitors that target the amyloid proteins participating in the cross-seeding events. The future scopes and major challenges in understanding the mechanism and developing therapeutics are also considered. Detailed knowledge of the amyloid cross-seeding will stimulate further research in the practical aspects and better designing anti-amyloid therapeutics.

## 1. Introduction

Amyloids are the aggregates of proteins that are insoluble and resistant to degradation. The formation of amyloids is generally associated with diseases collectively known as amyloidosis, though some amyloids do have functional roles Castellano and Shorter [1,2,3]. Several protein misfolding diseases (PMDs) are associated with the presence of amyloids, which are considered to be the hallmark of these diseases. For example, Parkinson’s disease (PD) is characterized by the presence of α-synuclein (α-syn) deposits, Alzheimer’s disease (AD) is characterized by the presence of amyloid-beta (Aβ) and tau plaques, and type-2 diabetes (T2D), apart from insulin resistance, is also characterized by the presence of human islet amyloid polypeptide (hIAPP) amyloid fibrils [4]. Structurally, significant conformational variability is seen in the amyloids formed by different proteins; however, they are predominantly composed of β-sheet secondary structures in a characteristic cross-β conformation stabilized by intermolecular hydrogen bonding [4,5]. The amyloid β-sheets can be arranged both in parallel [6,7] or antiparallel [8,9] orientations. Amyloids are also characterized by their physical features, such as the ability to bind with the dye Congo Red, resulting in apple-green birefringence in polarized light, and their ability to bind to fluorescent stains thioflavin-T and -S [10].

The rate-determining step in the formation of fibrils of misfolded proteins is the formation of “seeds”. Seeds are stable nuclei composed of polymerized proteins that can promote fibril formation by converting soluble proteins to fibrils [11]. The process of cross-seeding can either be homologous, i.e., seeds of the same protein, or they can be heterologous, i.e., seeds of one protein catalyzing the fibrillation of a different protein [12]. Cross-seeding aggregation between different amyloid proteins has been proposed to explain the presence of more than one misfolded protein in one disease and the coexistence of more than one PMD in the same individual [13,14]. It has also been observed that individuals diagnosed with one PMD are more susceptible to developing another [15,16]. The mechanism by which two different peptides form amyloids is not well understood. However, recent evidence suggests that heterotypic interactions between proteins via aggregation-prone homologous segments may contribute to it [17].

The terms cross-seeding and coaggregation are related but distinct. In the coaggregation process, two or more monomer protein influence the aggregation of each other independently of seeds, whereas in cross-seeding, the monomer or aggregate of one protein act as a seed for the aggregation of another protein. In the case of coaggregation, two proteins can polymerize together to form a mixed aggregate or fibrils or polymerize separately into distinct aggregates or fibrils.

## 2. Structure of Amyloids

Amyloids are the aggregates of protein that form long and unbranched fibers characterized by β-sheet structures in which the individual strands are arranged perpendicular to the axis of the fiber, forming a cross-β structure [18]. The cross-β pattern is considered to be the hallmark of the amyloid structure. The individual fibril, as visualized by transmission electron microscopy (TEM) and atomic force microscopy (AFM), are typically 7–13 nm in width and of the order of a few nanometers to micrometer in length [18,19]. X-ray diffraction studies have shown two characteristic scattering diffraction signals at 10 Å and 4.7 Å that correspond to the intersheet (stacking) and interstrand distance, respectively [18,19]. Amyloid fibrils are generally composed of substructures known as protofilaments [20,21]. These protofilaments vary in number and diameter and are often observed to twist around one another to form the 7–13 nm wide mature amyloid fibrils [4,5]. Each protofilament possesses the cross-β structure formed by variable numbers (typically 1–6) of sheets stacked on each other. The aggregation of a single amyloid protein can give rise to different structures. The amyloid polymorphism can arise due to differences in the arrangement, number, and packing of protofilaments. Solid-state nuclear magnetic resonance (ssNMR), a technique widely used for studying the structure of amyloids, was performed on amyloid fibrils of Aβ to show that β-sheets within the protofilaments can be arranged in parallel or antiparallel orientations [4]. Additionally, the ssNMR studies on Aβ and α-syn have revealed that the amyloid fibrils display in-register parallel β-sheet structures [22,23,24]. Cryo-electron microscope (cryo-EM) is another tool used to study high-resolution structures of amyloid fibrils. Cryo-EM studies of two types of amyloid fibrils- the paired helical filaments and straight filament, taken from the brains of patients with AD, revealed characteristic longitudinal cross-over distances ranging from 650–900 Å and filament widths ranging from 100–150 Å [25]. Another novel amyloid filament of tau, isolated from patients with chronic encephalopathy, revealed the presence of a more open conformation within the β-helix region than filaments observed in AD [26]. Recent cryo-EM studies on α-syn fibrils have shown the presence of two types of amyloid polymorphs—rod and twister structure [27]. The advancement in the use of multiple techniques to study the structure of amyloids associated with different diseases helps to understand the PMDs better and further the therapeutic and drugs development.

## 3. Intrinsically Disordered Proteins and Their Role in Amyloid Formation

Intrinsically disordered proteins (IDPs) lack a definite three-dimensional (3D) structure. The discovery of IDPs confronted the classical sequence–structure–function paradigm of proteins, which states that the 3D structure is responsible for its function. IDPs are flexible and exist in different conformational ensembles. They are rich in disorder-promoting amino acids such as Pro, Gly, Arg, Gln, Glu, Lys, Ser, and Ala and lack order-promoting amino acids such as Cys, Tyr, Trp, Val, Ile, Asn, Phe, and Leu [28,29]. IDPs have low hydrophobicity and high net charge, which promotes disorderedness due to strong electrostatic repulsion from the charged residues [30]. Many IDPs undergo a disorder-to-order structural transition upon binding to other biomolecules or can transform into “fuzzy complexes”, i.e., they remain partially disordered even when bound to another binding partner [31]. The amyloid-forming proteins such as Aβ, α-syn, tau, prion protein (PrP), etc. are partially or fully disordered in their native monomeric form [24,32,33,34,35,36]. The unique structural flexibility of these proteins allows for their aggregation through conformational polymorphism and oligomerization. The aggregation kinetics of amyloidogenic IDPs follows the typical nucleation-dependent polymerization. Many lines of evidence have shown that the soluble oligomeric species have disordered conformations and are more toxic than the matured fibrils [24,32,33,34,35,36]. Another feature of the amyloidogenesis and protein misfolding of almost all folded proteins is partial unfolding, which exposes the disordered region before the initiation of amyloid formation [37]. In globular proteins, the amyloidogenic sequence segments are buried within the core of the protein and, therefore, undergo partial unfolding before the formation of aggregates. For example, the conversion of α-helix to β-sheet in PrP is thought to occur due to helix-to-disorder transition. Additionally, the truncation of the C-terminal region of PrP, which is associated with diseases, yields a disordered variant that can readily undergo conversion into pathological amyloids [38,39]. However, besides their role in pathological amyloids formation, IDPs are primarily involved in a diverse range of biological functions such as cell division, cell cycle control, transcription and translational regulation, cell signaling, chromatin remodeling, and so forth [40,41]. Therefore, the aggregation of IDPs into amyloid fibrils has functional implications in a wide variety of organisms ranging from bacteria to humans [3,42,43].

## 4. Mechanism of Protein Aggregation and Amyloid Formation

The formation of amyloid fibrils from functional proteins involves a process of polymerization that is nucleation-dependent (NDP), resulting in the formation of β-sheet structures that are resistant to degradation and have a tendency to form larger aggregates [44,45,46]. The process starts with a slow nucleation phase, followed by an elongation phase, and ultimately ends in the saturation phase [44]. During polymerization, the rate-determining step is the formation of polymerized proteins, i.e., seeds. These seeds can then facilitate the further formation of amyloid fibrils [11]. A detailed description of the process is given in Section 6.4, with hIAPP as an example. In contrast to NDP, protein aggregation also occurs via isodesmic or linear polymerization, where the lag phase and critical monomer concentration are absent, which are the typical characteristics of NDP [47]. The kinetics of protein aggregation in linear polymerization does not require a separate nucleation and elongation rate, and therefore, the rate constants are identical for all the association steps.

A large number of studies suggest that the oligomeric intermediates that are formed during the aggregation process are toxic, and therefore, their interactions with cell membranes lead to cellular damages and cell death. The exposed hydrophobic regions in the oligomers seem to be responsible for their toxicity. Oligomers of the same size and morphology, but different exposed hydrophobic residues have variable toxicity levels [48]. The size of the oligomeric species is also responsible for toxicity, i.e., the smaller the size of the oligomers, the greater the toxicity it exhibits [48]. This could be due to the rapid diffusion of small oligomers within the cell, and hence, their interaction with different substrates could lead to cellular dysfunction. For instance, it has been shown that the toxic oligomers of Aβ and α-syn are responsible for the cellular damages in AD and PD, respectively [49,50]. Therefore, the toxicity of amyloids might be due to the interaction of oligomeric species with cellular compartments such as phospholipids, RNA, protein receptors, etc.

## 5. Seeding of Amyloid Proteins

**Figure 1:**
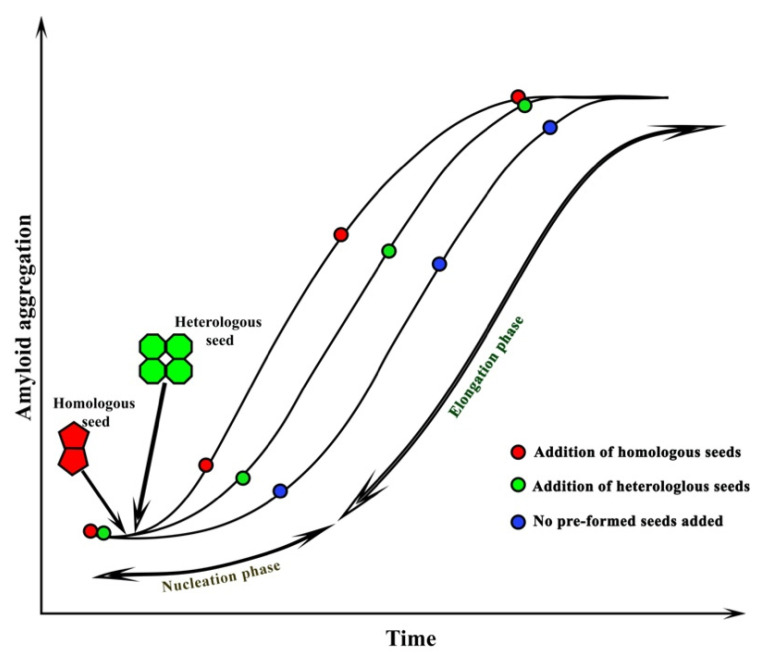
**Amyloid seeding and aggregation**. The addition of preformed seeds reduces the lag phase leading to faster aggregation. The seeds can be homologous or heterologous. Homologous seeds, which have the same nature as the existing nuclei, lead to homologous seeding, whereas the heterologous seeds differ from the initial nuclei and lead to heterologous or cross-seeding.

The slow nucleation phase can be accelerated by the addition of preformed amyloid fibrils (seeds) (Figure 1). This phenomenon is known as seeding. Seeding can be either homologous or heterologous [12]. Homologous seeding occurs when the preformed amyloid fibrils catalyze the aggregation of the same protein. In contrast, heterologous seeding, also known as cross-seeding, occurs when the polymerization of one protein is catalyzed by a different protein [12]. Numerous studies suggest that the interactions between different amyloids are observed in many neurodegenerative diseases [51]. These studies support the idea that cross-seeding interactions between different amyloid proteins are perhaps the reason behind abnormal protein aggregations found in different PMDs. The process of cross-seeding polymerization is similar to that of homologous seeding. During the process, the unstructured monomers are converted into semistructured seeds and finally into amyloid fibrils that are composed of β-sheets [52]. However, cross-seeding polymerization is more complex than homologous-seeding due to the presence of different proteins. Additionally, unlike homologous seeding aggregation that always occurs, not every two different amyloid proteins can cross-seed each other, suggesting the existence of cross-seeding barriers. Several studies have shown that only a few pairs of amyloid protein can promote amyloid aggregation and fibrillization. These include the microtube-associated protein tau (MAPT, tau) and Aβ [53], Aβ and α-syn [54], tau and α-syn [55], Aβ and hIAPP [56], Aβ and scrapie-associated prion protein (PrP^SC^) [57], and rat islet amyloid polypeptide (rIAPP) and hIAPP [58].

## 6. Different Types of Amyloids Forming Proteins

Many proteins enter the amyloid formation stages and end up forming elongated fibers with the backbone spine composed of β-sheets. As of today, there are over 25 amyloid proteins that have been identified to form amyloid and are associated with diseases. The most common amyloid proteins are Aβ, tau, α-syn, hIAPP, and PrP, which can also cross-seed among themselves.

### 6.1. β-Amyloid

**Figure 2:**
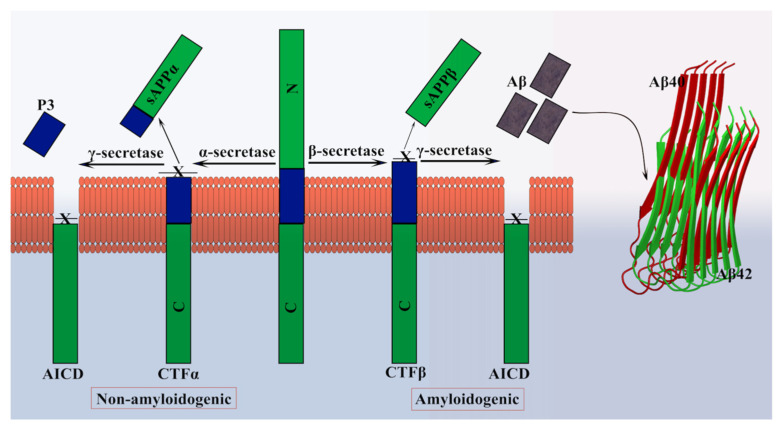
**Human APP cleavage pathway.** The human APP undergoes proteolytic cleavage in two different pathways: amyloidogenic and non-amyloidogenic. In the non-amyloidogenic pathway, the α-secretases cleave within the Aβ domain to form the α-C terminal fragments (CTFα), and the N-terminal soluble APP (sAPPα). The CTFα is subsequently cleaved by γ-secretase to form P3 and APP intracellular domain (AICD). In the amyloidogenic pathway, the β-secretase initially cleaves APP to form β-C-terminal fragments (CTFβ) and N-terminal-soluble APP (sAPPβ). The CTFβ is then cleaved by γ-secretase to form extracellular Aβ and AICD. The arrangement of Aβ40 (red) (PDB ID: 6TI5) [65] and Aβ42 (green) (PDB ID: 2BEG) [66] in the fibrillar phase are shown on the right. The β-sheet structure of the peptide and the parallel direction can be observed in the figure.

β-amyloid (Aβ) is a small peptide derived from a larger protein called amyloid precursor protein (APP). The human APP has two pathways for processing: non-amyloidogenic (α-secretase) and amyloidogenic (β-secretase and γ-secretase) (Figure 2). Even though the function of the APP protein remains elusive, the protein extends from the inside of a brain cell to the outside and passes through the fatty membrane that lies around the cell. In addition, the protein regulates synapsis formation and repair, anterograde neuronal transport, and iron export [59,60,61]. Aβ is a 4 kDa peptide and was first isolated from the amyloid deposits in the brain and cerebrovascular regions of the patients with AD and Down’s syndrome [62,63,64]. Aβ is formed when APP is proteolytically cleaved, and Aβ accumulates, resulting in the formation of senile plaques. The peptide is cleaved from the APP by two membrane-bound endoproteases: β-secretase and γ-secretase. Initially, the β-secretase cleaves the APP protein to release the soluble APP (sAPPβ), which is then sequentially cleaved by γ-secretase, to generate the Aβ. The γ-secretase cleavage is imprecise and, therefore, creates heterogeneity in the C-terminal end of the peptide. Structurally, the Aβ peptide contains a set of β-sheets that are parallel to the axis of the fibrils and extended strains perpendicular nearly to the fibril axis. The β-sheets are either parallel or antiparallel, and the sheets are aligned on top of one another in the fibril axis, i.e., they are “in register” (Figure 2) [34].

The multimeric assembly of the Aβ peptides is essential for their biological effects. Two phases of the assembly are known, and they vary in their characteristics and biological properties. Earlier, the amorphous and fibrillar deposits of Aβ were the point of focus but later shifted to the multimeric soluble forms of the peptide. The multimeric forms are much more toxic to cells than the fibrillar ones and can trigger several toxic events inside the cells [67]. Researchers argue that the soluble multimeric/oligomeric forms are active biologically and cause toxic effects in the cells; however, the mechanism of action remains elusive [68]. Structurally, both the phases are distinct to such an extent that the antibodies against multimeric forms do not recognize the monomer or fibrils and vice versa. The Aβ42 peptide readily undergoes oligomerization, while the Aβ40 forms, though abundant, do not oligomerize significantly [69]. The C-terminal region of the Aβ42 peptide is critical for the oligomerization process, and the ratio of Aβ42/40 is vital, as there lies a relation to the onset of the AD [70].

### 6.2. Tau Protein

**Figure 3:**
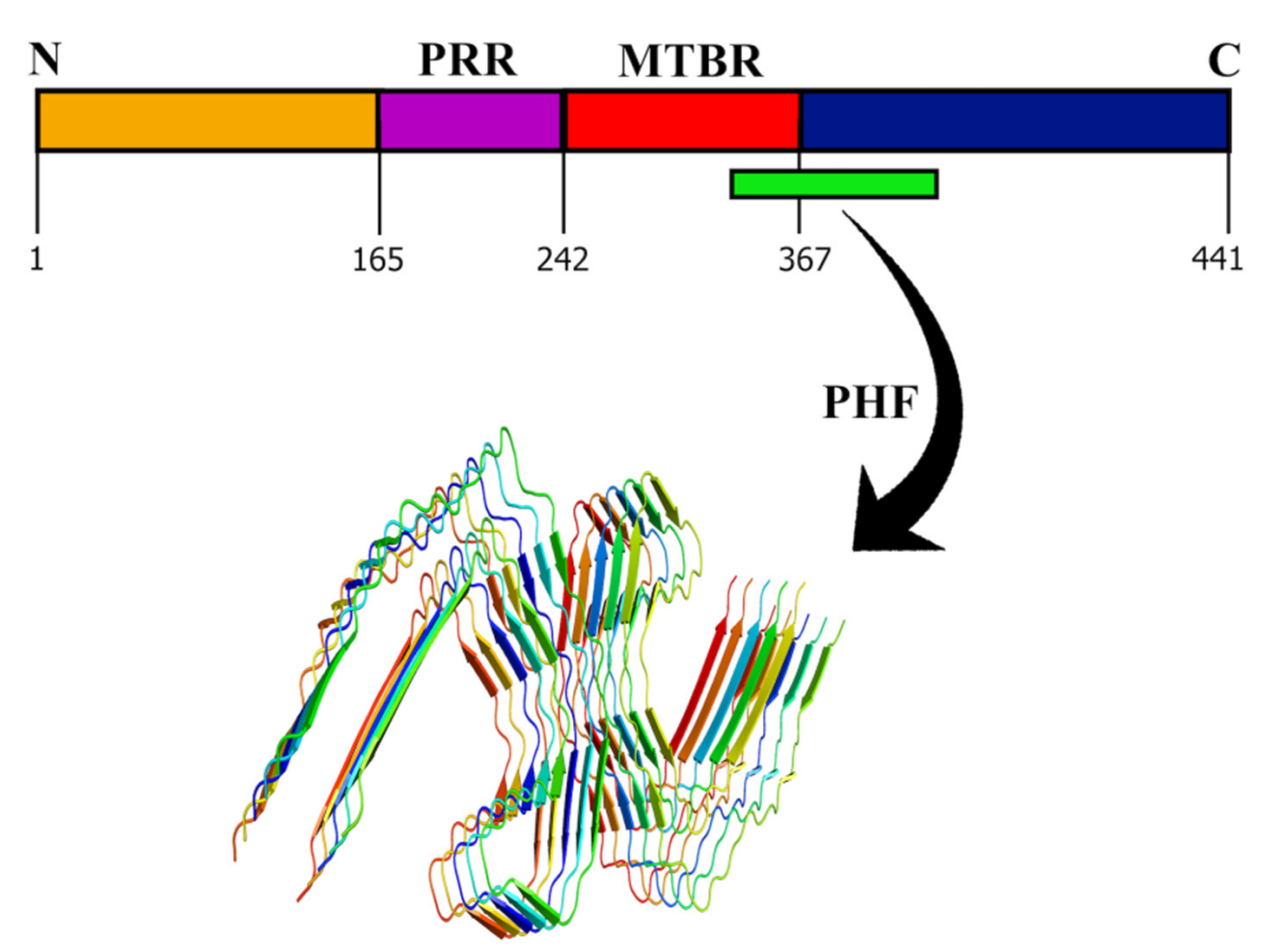
**Domain structure of tau protein.** The N-terminal domain, proline-rich region (PRR), microtubule-binding region (MTBR), and C-terminal region (CTD) are shown. The PHF aggregate from 308–380 amino acids (in green) of tau protein solved by cryo-EM (PDB ID: 7NRQ) [91] shows the assembly of tau in the diseased fibril phase.

Tau is another protein that contributes to the progress of AD and other neurodegenerative diseases such as dementia. The human tau protein is encoded by the microtubule-associated protein tau (*MAPT*) gene located on chromosome 17q21 [71,72]. The protein is found mainly in the axons of the central nervous system (CNS) and has six different isoforms generated by alternative splicing [73]. The proteins differ in the presence or absence of 29 amino acid repeats at the N terminal encoded by the exons 2 and 3 and by a 31 amino acid repeat in the C terminal. Structurally, tau protein has an N-terminal domain (1–165), followed by a proline-rich domain (166–242), a microtubule-binding region (MTBR) (243–367), and a C-terminal domain (368–441) (Figure 3) [74].

Tau is a vital microtubule-associated protein in the brain; however, several other MAPs have also been discovered [75]. The reason behind the limelight on tau is its association with AD. It is established that tau is required for the induction of Aβ toxicity [76]. The mechanism has been attributed to the hyperphosphorylation of the tau protein at several sites (eight or more phosphates per tau molecule) [77]. This can be due to several other physiological conditions and, therefore, should not be considered an indicator of AD onset [78]. Most of the phosphorylation sites in tau are in the proline-rich region and the C-terminal domain, except S262, S293, S324, and S356 [79,80,81]. The diseased condition might be either due to the upregulation of tau kinases or the downregulation of tau phosphatases [80,82]. The abnormally phosphorylated tau is incapable of binding to the tubulin and cannot promote microtubule assembly. On the other hand, it has also been known to inhibit assembly and disrupt the organization of microtubules [83,84]. Apart from hyperphosphorylation, another suggested mechanism is the acetylation of tau that leads to AD and other neurodegenerative disorders. The acetylation of K280 leads to the loss of capacity to bind to microtubules. This dysfunction causes paired helical filaments (PHF) aggregation and increased pools of cytosolic tau [85,86]. The typical arrangement of PHF is shown in Figure 3. It was also found that acetylation of tau was associated with hyperphosphorylation, indicating that tau dysfunction could be the cause of acetylation and hyperphosphorylation independently or in combination [87]. An alternative mechanism to the abnormal functioning of tau has been proposed to be due to proteolytic cleavage of tau [88]. The components of the PHF core are composed mainly of MTBR, which is truncated at E391 (C terminal), but the enzyme responsible remains unexplored [89]. Apart from AD, tau is also involved in other diseases such as Pick’s disease (straight filament (SF) aggregation), chronic traumatic encephalopathy (hyperphosphorylated tau with PHF and SF), and corticobasal degeneration (hyperphosphorylated tau) [90].

### 6.3. α-Synuclein

**Figure 4:**
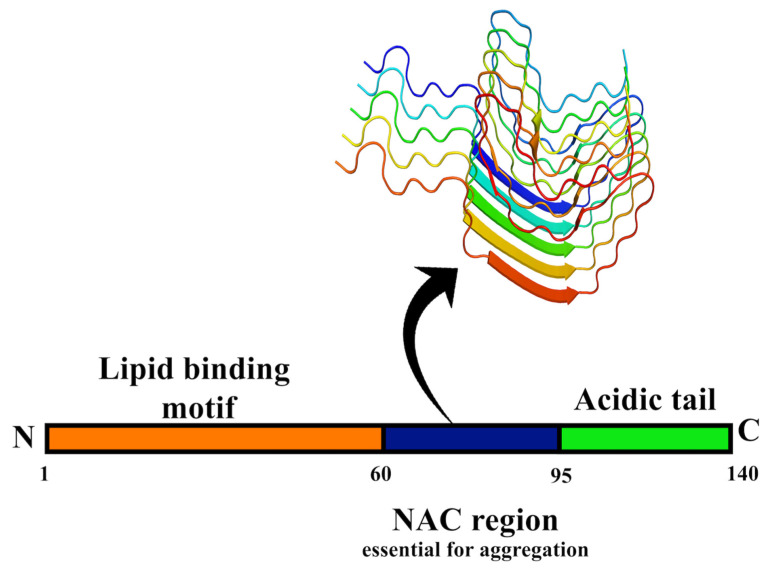
**Domain structure of the α-syn protein.** The lipid-binding N-terminal domain (NTD) is represented in orange, while the non-Aβ component (NAC) region necessary for the aggregation of α-syn protein is in blue. The C-terminal domain (CTD) of α-syn protein is represented in green. On top, the arrangement of α-syn protein in fibrillar form is shown (PDB ID: 6FLT) [27].

α-synuclein is a 140-amino-acid-long protein encoded by the *SNCA* gene. It is linked to diseases such as multiple system atrophy, AD, dementia, brain iron accumulation, neurodegeneration (type I), pure autonomic failure, and tremors [92]. The protein does not possess a defined structure in an aqueous solution and is, therefore, termed a natively unfolded protein. Interestingly, when α-syn binds to negatively charged lipids, it forms α-helix, and on prolonged incubation, it can also form β-sheet rich structures. There are two other members in the synuclein family of proteins: β-synuclein and γ-synuclein. They differ from each other in the central hydrophobic domain and localize preferentially. The protein comprises three unique domains—namely, (1) the N-terminal domain (NTD, 1–60) with lipid-binding motifs, which forms α-helices on membrane binding (amphiphilic helices); (2) a central hydrophobic domain (61–95), also called non-Aβ component (NAC), which confers β-sheet potential and contain minimal sequence required for aggregation; (3) a C-terminal domain (CTD, 96–140), which is highly unstructured, anti-amyloidogenic, and involved in Ca^2+^ binding (Figure 4) [93,94].

α-synuclein is capable of forming β-sheets under certain conditions, and this generated considerable interest, as it was similar to the β-sheets formed by Aβ. It was later found that the formation of β-sheets by α-syn is a result of the pathogenesis of neurodegenerative diseases. The mutations in α-syn induce the formation of amyloid-like fibrils, which has been observed only on prolonged incubation. The most common mutations are A53T and A30P, which forms protofibrils. The A53T mutant readily forms mature fibrils, and E46K formed fewer protofibrils when compared with wild-type α-syn [95,96]. Several other mutations are reported in the *SNCA* gene to cause PD, such as H50Q, G51D, A53T, A53E, A53V, and A30P. The mutations might increase the aggregation rate, a change in oligomerization and conformation, or a decrease in the tetramer/monomer ratio [97]. The α-syn undergoes multiple post-translational modifications (PTMs); the best-studied ones are phosphorylation. Earlier, it was believed that the soluble oligomer formation might be due to phosphorylation as observed in tau proteins [98,99], but later, it was shown that the effect of phosphorylation is contradictory. The phosphorylation of S129 occurs minimally in normal physiological conditions. The increased expression of α-syn phosphatases reduced the α-syn mediated neurotoxicity exhibited earlier [100].

### 6.4. hIAPP

Human islet amyloid polypeptide (hIAPP) is an amyloidogenic protein secreted as a randomly unstructured peptide. It plays a vital role in the progression of type 2 diabetes mellitus (T2DM). The autopsies of bodies with T2DM displayed hIAPP aggregates in the pancreatic islets. This was in line with the reduced pancreatic islets functioning and β-cell mass reduction, which, in turn, caused impaired insulin secretion [101]. Though the peptide is initially secreted with a random structure, it later assumes conformations such as helical, cross-β-sheets, and β-sheets, before it transforms into the amyloidogenic aggregated stage.

**Figure 5:**
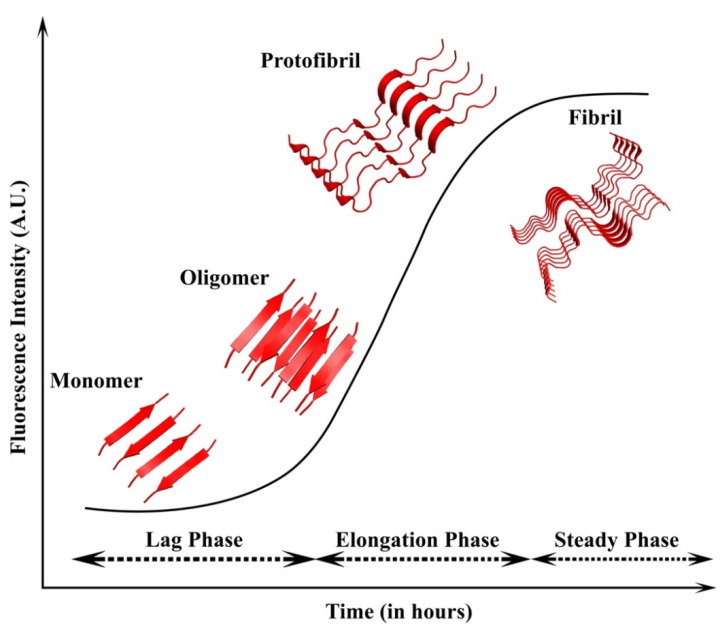
**Schematic representation of hIAPP aggregation kinetics.** The sigmoidal curves house the different forms: monomer, oligomer, protofibrils, and fibrils of hIAPP that are formed at various phases with time. The representation is shown for kinetics without seeding. The structures of different forms of hIAPP are shown (PDB IDs: 2KIB and 6VW2) [110,111].

IAPP belongs to the calcitonin family comprising adrenomedullin, α- and β- calcitonin gene-related peptide (CGRP), calcitonin, and intermedin. All the peptides undergo extensive PTMs. Interestingly, IAPP shares sequence similarity with CGRP but shows a divergence in the 20–29 amino acid segment [102]. Moreover, hIAPP is amyloidogenic in nature, while CGRP does not form amyloid. Later, it was shown that the 10-residue region of hIAPP is responsible for the aggressive amyloidogenic property [102,103]. The residues V17, N22, and N23 in hIAPP were essential for the amyloid formation, and the 22–25 residue segment provided the nucleation template for the formation of fibrils [104]. The formation of fibrils is divided into three stages. Initially, the primary nucleation occurs, during which the monomeric peptides come together to form small oligomeric peptides that are soluble. These small oligomeric peptides form the critical nuclei that are energetically unstable, causing further amplification and growth [105]. This is followed by the elongation phase, in which the protofibrils propagate, and the monomer consumption and fibril growth occur [106]. This step is entropically favorable and results in the steady-state or plateau phase (Figure 5). In this phase, the concentration of fibrils dominates, along with equilibrium with the monomer concentration [107]. Apart from this, mutations have also been known to fasten the aggregation kinetics. Point mutations such as R18H, L23F, V26I in rat IAPP exhibited more rapid aggregation than the wild type [108]. The S20G mutation also displayed rapid aggregation, which was attributed to the smaller size of glycine when compared with serine, which might help in fibril packaging [109].

### 6.5. PrP Protein

Prion diseases cause some of the deadliest neurodegenerative diseases, affecting the motor and cognitive functioning of animals, including humans. They are caused by infectious proteinaceous particles called prions (PrP) [112,113]. PrP is a ubiquitous glycoprotein anchored to the plasma membrane of cells with the help of a glycosylphosphatidylinositol (GPI) anchor [114,115]. The protein is highly conserved in mammalian cells. The prion diseases include spongiform encephalopathy in cattle, scrapie in sheep and goats, chronic wasting in deer and elk, and kuru, fatal familial insomnia, Gerstmann–Straussler–Scheinker disease, and Creutzfeldt–Jakob disease (CJD) in humans [116]. Though the PrP protein has been studied, the precise function of the protein remains uncharacterized. The misfolding of the cellular prion protein (PrP^c^) results in the pathogenic isoform (PrP^sc^), which causes neurodegenerative prion diseases. Though PrP^sc^ has been deciphered to be the causative agent in the related diseases, this is not always the case, as the absence of PrP^sc^ has also displayed prion diseases. Therefore, other molecular species of PrP^c^, different from PrP^sc^, might also be the primary neurotoxic components. One such example is PrP^ctm^, a transmembrane form of PrP, which is detected in the brains of human prion disease patients [117]. Therefore, it is imperative to understand the function, localization, and trafficking of the proteins that cause prion diseases. The function of the PrP protein has been studied to be the metabolism of copper, an essential metal cofactor required in various enzymes in the human nervous system [118]. The protein can reduce Cu^2+^ to Cu^+^ and decrease the formation of reactive oxygen species. In addition, the PrP protein is involved in cell–cell adhesion [119].

**Figure 6:**
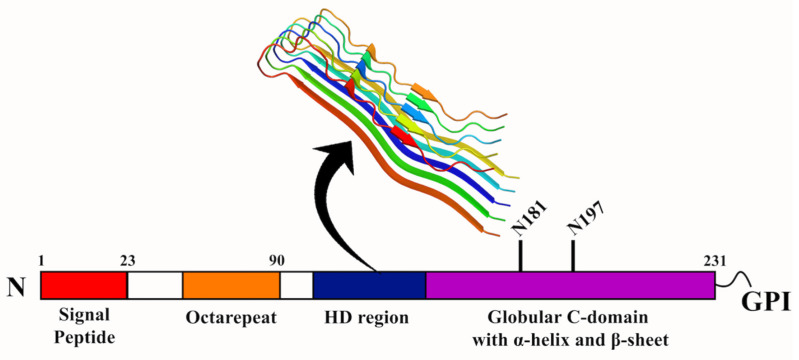
**Domain structure of PrP protein.** The signal peptide that guides the PrP protein is represented in red, with the octapeptide in orange. The globular C-terminal domain (CTD) with α-helices and β-sheets are shown in purple. The hydrophobic domain (HD) between the octapeptide and the CTD is colored in blue. The fibril form of human PrP is shown at the top (PDB ID: 6UUR) [126].

The PrP protein is synthesized from a single gene that codes for 250 amino acids. The N-terminal region of the protein houses five proline residues, followed by glycine-rich octapeptide repeats capable of binding copper. The hydrophobic domain (HD), which is the central segment of the PrP protein, is highly conserved and hydrophobic. The protein also contains a disulfide bridge and two glycosylation sites. The C-terminal region is hydrophobic and contains the signal peptide for GPI- anchor attachment. The important signal peptide in PrP^c^ is the N-terminal signal peptide of 22 amino acids, which is recognized by signal recognizing particles. Therefore, the integrity of the N-terminal signal peptide is crucial for the import and export of PrP^c^ [120]. The most important feature of PrP^c^ is the endoproteolytic processing by two internal cleavage processes, i.e., α-cleavage and β-cleavage. In a healthy environment, PrP^c^ is cleaved between amino acids 110/111 by an α-cleavage process resulting in a 17 kDa C-terminal fragment (C1) and 9 kDa N-terminal fragment (N1). These two fragments are the products of prion metabolism and increase PrP^c^ dimerization. Under the diseased condition, β-cleavage of PrP^c^ at amino acids 90/91 generates a 21 kDa C2 fragment, and this transition from C1 to C2 leads to the PrP^sc^ propagation. The inefficient import of PrP protein to the ER results in its abnormal accumulation, thereby interfering with the cell viability [121]. Apart from the N-terminal region, the HD region (106–126) holds prominence in that it carries amyloidogenic properties and can form fibrils in vitro and produce toxic effects in cultured cells (Figure 6). Curiously, transgenic mice that expressed PrP lacking HD region could not develop transmissible prion infection, suggesting that the HD region was linked to the toxic effects exhibited by the PrP protein [122,123,124]. Furthermore, spectroscopic studies have identified that the PrP^c^ and PrP^sc^ differ conformationally in that the PrP^c^ contains high α-helical content (~42%), with no β-sheets, while PrP^sc^ comprises 30% of α-helix and 45% of β-sheets in its structure [125].

Transmissible spongiform encephalopathy (TSE) is another fatal neurodegenerative disorder caused by PrP^c^. The disorder results from the misfolding of PrP^c^ to its scrapie isoform PrP^sc^. The PrP^sc^ isoform is insoluble, partially protease-resistant, and can propagate by interacting with the normal isoform PrP^c^. PrP^sc^ has a strong polymerizing tendency, and as a result, it forms amyloid aggregates. These aggregates are usually observed to be accumulated in the brain [120]. The accumulation is generally observed in the lymphoreticular region and brain, and the effect is typically neuronal vacuolation and death. Though the involvement of PrP protein in neurodegenerative disorders is known, the mechanism of conversion is still elusive. There are several hypothetical models, and they largely remain unexplored [127,128,129,130].

## 7. Cross-Seeding of Amyloid Proteins: Role and Mechanism

Amyloid-forming proteins aggregate independently to cause several neurodegenerative diseases. However, recently, these amyloids have been discovered to undergo cross-seeding. Cross-seeding is a biological event wherein the amyloid structures of one type of protein (homologous amyloids) can act as a seed and facilitate the aggregation of another amyloid protein, forming heterologous amyloids. Similar to homologous aggregation, the process of cross-seeding follows the same steps of nucleation, elongation, and the plateau phase (Section 6.4) [46,131,132]. Cross-seeding follows the nucleation-dependent aggregation pathway and requires a template to assist growth [133]. The nucleation phase is critical where the monomeric protein, in either mutated, denatured, or an oligomeric state, aggregates to overcome the high energy barrier. It is in the growth phase that various amyloid proteins come together in different forms to form deposits. The cross-seeding aggregation differs from the spontaneous aggregation in that the lag phase is reduced, and the aggregation kinetics is considerably faster in cross-seeding than in self-seeded aggregation [46,134]. Additionally, the aggregation in cross-seeding results from intermolecular interaction between different proteins, especially oppositely charged hetero-proteins [135], which contrasts to homologous amyloids, in which similarly charged proteins are electrostatically repulsed. The heterologous amyloids provide an electrostatically favorable environment and exposition of partially hydrophobic surfaces. These hydrophobic surfaces further trigger the nucleation and growth of aggregates [135]. However, not all two amyloid proteins can cross-seed each other, suggesting the existence of a cross-seeding barrier. Two models have been proposed for the mechanism of cross-seeding: template-assisted growth model and conformational and selection shift model. In the template-assisted growth model, depending on the aggregation and folding kinetics of different amyloids, the one that can form more populated seeds serves as a template and recruits the other amyloids for aggregation. In the other model, if both the amyloids have similarly populated seeds, the structural equilibrium lowers the barrier and selects those heterologous seeds with high conformational similarity, leading to cross-seeding [52]. A list of cross-seeding of various amyloid proteins deduced via multiple experimental and computational approaches is provided in Table 1 [52].

### 7.1. Cross-Seeding of Aβ and Tau

As discussed earlier, Aβ peptide found in plaques occurs in several forms, and studies have revealed that the different versions of Aβ interact with each other [160,161,162,163]. Apparently, cross-seeding has been observed between Aβ40 and Aβ42 peptides in in vitro conditions [161]. Apart from plaques, another hallmark of AD is the deposition of neurofibrillary tangles (NFTs), which mainly contain tau proteins [87]. Usually, Aβ plaques are deposited extracellularly, while the NFTs are formed intracellularly [164], but several pieces of evidence point toward the interaction between Aβ and tau protein. In vivo studies have shown that the Aβ species can accelerate the formation of NFTs [165] and form stable complexes with tau species [166]. The amyloid core sequence KLVFFA and the C-terminal residues of Aβ bind to tau [166]. The tau segments VQIINK and VQIVYK, located at the beginning of repeat 2 (R2) and repeat 3 (R3), respectively, of the four microtubule-binding repeats K18, can bind Aβ [167]. The peptides from regions of tau in exon 7 and 9 can also bind to Aβ [166]. Through the computational seeding model, it was predicted that the amyloid core of Aβ can form intermolecular β-sheet interactions with VQIINK or VQIVYK [143]. Recently, it has been reported that the peptide-based inhibitors of Aβ could reduce the aggregation and self-seeding of tau fibrils [168]. The ability of the inhibitors to interfere with the aggregation of both Aβ and tau suggested that both proteins share a common binding region. This supported the hypothesis that the interaction is through the cross-seeding mechanism [168,169].

### 7.2. Cross-Seeding of Aβ and α-Syn

The cross-seeding in α-syn has been observed between the isoforms of α-syn such as SNCA140, SNCA126, SNCA112, and SNCA95 [170,171]. Using in vitro experiments, it was shown that the C-terminal-truncated form of α-syn can seed the full-length form, leading to the formation of the Lewy body. Lewy body aggregates were also formed when the synphilin-1 protein interacted with α-syn [172,173]. The synphilin-1 A protein can interact with α-syn and synphilin-1 and cause aggregation [174]. The aggregation of α-syn into Lewy bodies (LBs) and Aβ into amyloid plaques are associated with PD and AD, respectively. LBs containing α-syn are usually found as aggregated intracellular vesicles [175], while Aβ is deposited extracellularly as senile plaques [176]; however, several studies have shown overlapping symptoms between the patients with AD and PD, suggesting a cross-talk between the two proteins. A study in transgenic mice demonstrated that Aβ could augment the aggregation of α-syn [177]. Conversely, α-syn has been shown to enhance the aggregation of Aβ both in vivo and in vitro. In addition, the non-amyloid component (NAC) region of α-syn was found in Aβ deposits in AD patients, indicating the interaction of Aβ with α-syn [178]. Moreover, employing nuclear magnetic resonance (NMR) spectroscopy in the membrane mimicking environment, it was shown that α-syn interacted more strongly with Aβ42 than with Aβ40 to produce more toxic oligomers [179].

### 7.3. Cross-Seeding of Aβ and IAPP

Several studies have shown that individuals with AD develop signs and symptoms of T2D or other glucose-related disorders, while individuals with T2D are at a higher risk of developing AD than healthy individuals. The exact mechanism behind the correlation of AD and T2D is still unknown, but multiple studies have indicated the cross-seeding interaction between Aβ and IAPP (amylin). A study on the interaction of Aβ and amylin had shown that hIAPP promoted Aβ42 oligomerization and the formation of larger aggregates [180]. It was also observed that Aβ42 and hIAPP interacted to form heterocomplex aggregates, which induced cell death in neuroblastoma cells [180]. In transgenic mice, intravenous injection with preformed Aβ fibrils triggered IAPP amyloid formation in the pancreas of the mice, suggesting that Aβ could enhance IAPP amyloid formation through cross-seeding [181].

### 7.4. Cross-Seeding of Tau and α-Syn

The coexistence of tau and α-syn proteins has been observed in many neurodegenerative diseases, indicating the interaction between these two proteins. Immunohistochemical examination of brains of Down’s syndrome patients has shown the coexistence of α-syn and tau in 50% of Down’s syndrome with AD patients [182]. In vitro studies using different cell models of synucleopathies have shown that tau can promote the aggregation and toxicity of α-syn [183]. In vivo studies using a mouse model have demonstrated that injecting α-syn oligomers derived from PD patients into Htau mice accelerated the formation of tau oligomers, along with neuronal cell loss [184]. It was observed that the coexpression of tau and α-syn in *Dictyostelium discoideum* had a positive effect on phagocytosis, growth, and respiration rate [185]. A study on the fruit fly model revealed the cross-seeding between tau and α-syn impaired the eyes and dopaminergic neurons [186], indicating a broad impact of cross-seeding. It has been reported that a simultaneous introduction of α-syn mouse preformed fibrils (mpffs) and AD lysate-derived tau seeds increased tau aggregation [187] Conversely, the absence of tau did not affect the aggregation of α-syn, showing that only α-syn can act as a seed for tau cross-seeding but not vice versa.

### 7.5. Cross-Seeding of α-Syn and IAPP

α-syn is the major aggregated peptide in substantia nigra neurons of patients with PD, while IAPP is the major peptide found in pancreatic beta cells; however, several studies have reported the presence of α-syn in the pancreatic beta cells [188,189,190]]. Recent studies on transgenic mice overexpressing hIAPP reported the colocalization of both α-syn and hIAPP in pancreatic beta cells of the transgenic mice, as well as in human pancreatic beta cells [191]. It was observed that α-syn promotes cross-seeding of hIAPP in a dose-dependent manner, both in vitro and in vivo [191]. It was also observed that injecting α-syn monomers exogenously in mice promoted faster aggregation of IAPP, whereas IAPP amyloid formation was reduced in mice lacking the gene encoding α-syn [191], further implying the cross-seeding interaction between α-syn and hIAPP. Recently, it was reported that the octapeptide TKEQVTNV from α-syn can cross-seed with hIAPP monomers and facilitate hIAPP fibrillation [192]. Moreover, the cross-seeding between the octapeptide from α-syn and hIAPP could increase cell viability and reduce cell apoptosis by reducing hIAPP induced cytotoxicity [192], suggesting a broader impact of cross-seeding.

### 7.6. Cross-Seeding in Prion Disease

Studies have revealed the coaggregation of prion proteins with other amyloid proteins. The Aβ42 extracted from the Alzheimer’s brain has been found to coaggregate with human prion proteins. The study suggested the presence of cross-interactions between the two proteins and also the diseases [193]. The coexistence of PD and prion proteins has also been shown in patients [194]. Curiously, it is claimed that the PrP^c^ protein could facilitate the uptake of α-syn amyloid protein inside the cell, indicating the presence of possible intermolecular interactions between the two amyloid diseases [194]. It was reported that the peptide sequence “GNNQQNY” from a yeast protein (Sup35) could cross-seed with both hIAPP and Aβ to form β-structure aggregates, which accelerated amyloid fibrillization [191].

Although prions are neurogenerative diseases that affect humans and animals, evidence suggests that prions cross the species barrier. This results in cross-seeding of the prion proteins, and there is a consensus on the transmission of bovine spongiform encephalopathy from cattle to humans. There are key points for cross-species seeding that include (1) the difference in physiology between humans and animals under question, (2) the difference between the amino acid sequence of the humans and that of the animal PrP^C^, and (3) the prion strain of the animal coded in the conformation of PrP^sc^.

The prion cross-seeding has been studied using the protein misfolding cyclic amplification (PMCA) procedure. The prion strain variation and polymorphism in the codon 129 of the *PRNP* gene are the major factors responsible for the clinicopathological phenotype and the susceptibility of an individual to develop prion disease. In 2000, Parchi et al. showed that codon 129 is the primary determinant of the proteinase cleavage site in the PrP^sc^ in CJD. A change in this codon leads to a change in the size of the PrP proteinase-resistant fragments [195]. The allelic variation percentage of this codon varies among different ethnic groups. One such case of M129V has been reported in the United Kingdom [196]. A recent study revealed the association between PRNP M129V polymorphism and mild cognitive impairment and dementia, including AD, in a Rotterdam-based population study [197]. Methionine homozygous individuals have a higher susceptibility to CJD, while codon 129 heterozygous individuals have a longer disease duration [198,199]. This is referred to as the variant CJD, predominantly found in the UK and affects younger people. On the other hand, the sporadic CJD is endemic throughout the world and affects patients of median age of 68 years. The two types have differences in symptoms, diagnosis, and incubation periods. Interestingly, Jones et al. studied the transmission properties of CJD using PMCA. The study used human brain tissues or transgenic mice models with three genotypes of codon 129 (MM, MV, and VV). The results showed that the MM genotype of codon 129 had strong PrP^sc^ infectivity and conversion while the MV genotype and VV amplification had lesser and no amplification, respectively [200,201]. Therefore, the *PRNP* codon 129 genotype affects the susceptibility and phenotype of CJD.

### 7.7. Cross-Seeding in Other Proteins

Huntingtin (Htt), a hallmark protein of Huntington’s disease, has been observed to cross-seed and promote the fibrillation of TIA-1, an RNA-binding protein rich in glutamine and asparagine residues [202]. Another study found the occurrence of polyQ inclusions along with α-syn in samples of brain collected from HD rat models [203,204]. Aβ aggregation was influenced by cross-seeding with unrelated proteins that share a homologous aggregation-prone segment [205]. One clinical example for cross-seeding is the amyloidosis transthyretin (ATTR), which leads to a toxic function gain. TTR is a protein produced in the liver for the transport of thyroxin and retinol [206]. In the disease, the ATTR assembles into amyloid fibers and causes systematic organ dysfunction. Liver transplantation has been recommended as a treatment for ATTR variant (ATTRv) amyloidosis. However, due to the shortage of liver donors, there has been transplantation of livers from ATTRv patients. Fascinatingly, ATTR has been reported in patients who had received livers from ATTRv donors, specifically the V30M variant [207,208]. This is referred to as acquired ATTR following domino liver transplantation [207]. These recipients report the systemic amyloid deposition even before the appearance of amyloidosis symptoms in the patients with a mean time of 8 years [208,209]. In addition, liver transplant has led to the rapid and continuous deposition in cardiac tissues caused by the addition of the ATTR wild-type to the amyloid. This also has been observed in some V30M patients with cardiac amyloidosis [210,211,212,213].

## 8. Dual Inhibition

**Figure 7:**
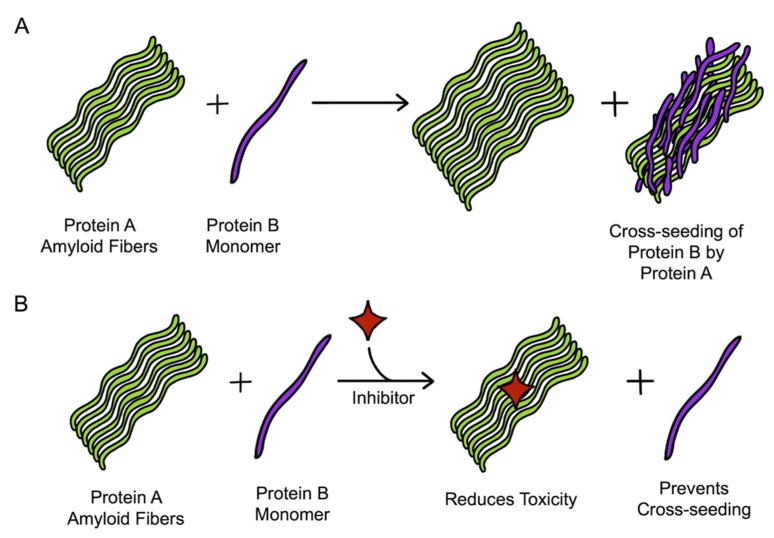
**General mechanism of dual inhibitors of cross-seeding:** (**A**) in the absence of an inhibitor, protein A acts as a seed and facilitates the cross-seeding of protein B; (**B**) in the presence of a dual inhibitor, the toxicity of the amyloid fibers of protein A is reduced, and the cross-seeding of protein B is also inhibited.

Cross-seeding of amyloid proteins has been observed to impact cell pathology [180,184,186], and therefore, targeting the involved amyloid proteins together might be a better therapeutic approach than individually targeting them [169]. Identification of the inhibitors having dual inhibition property is gaining the attention of many researchers. Many inhibitors, having dual inhibition effects against different pairs of amyloids aggregation, have been reported. The general mechanism of the working of dual inhibitors is depicted in Figure 7. We now discuss the available dual inhibitors of amyloid pairs involved in a few widely recognized PMDs.

### 8.1. Dual Inhibitors against Aβ and Tau

Several dual inhibitors against Aβ and tau have been reported. Recently, an extended in cellulo, in silico, and kinetic study was performed to test the inhibition efficiency of 1-benzylamino-2-hydroxyalkyl derivatives to identify a potent inhibitor against Aβ and tau [192]. It was observed that one of the compounds could inhibit 80% Aβ42 aggregation and 68.3% tau aggregation. The docking studies showed that the compound inhibited the aggregation process of Aβ by forming hydrophobic interactions, thereby stabilizing the α-helical structure of amyloid. The compound could inhibit tau fibrillization by binding to the central part of misfolded tau. The data from the docking studies also suggested the significant impact of chirality on the antiaggregation property of the inhibitor. The researchers suggested that S-isomers are favorable for Aβ and R-isomers for tau [192]. A series of peptide-based inhibitors have been designed that act as dual inhibitors against Aβ and tau [168]. The inhibitors could also reduce the efficiency of tau aggregation mediated by Aβ [168,169]. It has been reported that a curcumin derivative, PE859, could also act as a dual aggregation inhibitor against Aβ and tau in the mouse brains displaying the aging phenotype [214]. In addition, a furan coumarin (notopterol) has been identified to possess a dual inhibitory effect on β-secretase and GSK3β, the key enzymes responsible for Aβ production and tau phosphorylation, respectively [215]. The fact that these inhibitors could reduce the aggregation of both Aβ and tau further support the cross-seeding interaction between Aβ and tau. Recent in vivo studies on mice models have highlighted the neuroprotective properties of epigallocatechin-3-gallate (EGCG), a polyphenol constituent of green tea [216]. It was observed that treating the AD rats with EGCG decreased tau hyperphosphorylation in the hippocampus. Additionally, the expression and activity of Aβ42 and BACE1 were suppressed by EGCG, thereby improving the learning and memory function of AD rats. Methylene blue (MB) is another compound capable of reducing the aggregation of tau as well as Aβ. The ability of methylene blue to inhibit aggregated tau interaction with Aβ monomeric species in vitro through oxidation of cysteine residues serves it as a potent dual inhibitor against tau and Aβ [217]. Although MB reduces tau aggregation, it fails to act upon tau oligomers and thus showed poor performance in AD clinical trials [218].

### 8.2. Dual Inhibitors against Aβ and hIAPP

Numerous studies have reported that the potential link between AD and T2D could be due to the cross-seeding of Aβ and hIAPP [156,219,220,221]. Therefore, developing drugs targeting the cross-seeding between Aβ and hIAPP would be more effective than targeting the individual amyloids. Several inhibitors capable of inhibiting both the amyloids have been reported. Recently, bleomycin, a drug widely used as an antibiotic and antitumor, displayed a dual inhibitory effect on Aβ and hIAPP aggregation in vitro [222]. Genistein, a phytoestrogen in soybean, widely used as a cerebrovascular and anti-inflammatory drug, was also reported to have a dual inhibition effect on Aβ and hIAPP aggregation and increase cell viability and reduce cell apoptosis [223]. The polyphenol pentagalloyl glucose (PGG) has also been reported to inhibit the fibrillation of both Aβ and hIAPP [224,225]. PGG, at equal molar ratios to IAPP, was found to reduce fibril formation of IAPP [224]. In addition, tanshinones, the major component of the Chinese herb danshen (*Salvia miltiorrhiza* Bunge), could inhibit the aggregation of Aβ and hIAPP, disaggregate preformed hIAPP and Aβ fibrils, and also protect the cells from hIAPP and Aβ induced toxicity [226].

### 8.3. Dual Inhibitors against Aβ and α-Syn

Many AD patients develop signs and symptoms of PD and vice versa, indicating that the overlapping pathological pathways could be due to cross-seeding of Aβ and α-syn. Inhibitors have been reported to inhibit the aggregation of both Aβ and α-syn. Entacapone and tolcapone, anti-Parkinsonian drugs, could inhibit oligomerization and fibrillogenesis of both Aβ and α-syn in vitro and protect against the cytotoxicity induced by aggregation of both proteins as observed in the PC12 cell lines of rat adrenal gland pheochromocytoma [227]. Further, an in vitro study has shown that curcumin, the primary bioactive compound found in turmeric, has inhibitory effects against the aggregation of Aβ and α-syn [228].

### 8.4. Dual Inhibitors against Tau and α-Syn

The coexistence of aggregates of tau and α-syn in different pathologies has opened the doors to look for inhibitors that can act on both proteins, thereby preventing the formation of toxic aggregates. Recently, a small molecule (MG-2119) was identified as a potent dual inhibitor of monomeric tau and α-syn [229]. Using techniques such as cellular fluorescence resonance energy transfer, isothermal titration calorimetry, surface plasmon resonance, and microscale thermophoresis, the binding of the molecule to tau was investigated, and thioflavin T assay and dynamic light scattering results verified that it also inhibited the aggregation of α-syn. In SH-SY5Y neuroblastoma cells, the molecule reduced cytotoxicity in a dose-dependent manner.

## 9. Conclusions and Future Perspectives

Amyloid proteins can misfold and aggregate, causing disease conditions, and a few of them can interact homogenously and/or heterogeneously. Here, we attempt to shed light on the amyloid proteins, their cross-seeing behavior, and inhibition. Homologous aggregation of amyloid proteins has been extensively studied; however, the occurrence and mechanism of cross-seeding have not been extensively ventured. It is now accepted that cross-seeding is no longer an isolated but a well-established event in the growth of amyloid structures. Interestingly, the cross-seeding event is species-specific, and therefore, we discussed the aggregation of five hallmark amyloid proteins Aβ, αs, PrP^c^, tau, and hIAPP. It is hypothetically assumed that the cross-seeding between amyloids is dependent on conformations that lower the energy barrier for seeding. The mechanism of the formation of amyloid aggregates is still elusive for most proteins and requires a more profound understanding of anti-amyloid drug design and discovery. The knowledge of the protein components involved in the cross-seeding warrants further investigation to clarify the role and mechanism of cross-seeding and aggregation. The inhibitors targeting dually on amyloid proteins participating in the cross-seeding event inhibited heterologous aggregation but also caused disassembly of the aggregates. Understanding the molecular mechanism of interaction in cross-seeding will help develop better therapeutics against PMDs. Furthermore, researchers can strategize approaches to inhibit and disassemble both homologous and heterologous aggregates by designing site-specific inhibitors. Further exploration and in-depth studies on the amyloid cross-seeding will help gain more significant insights into understanding the amyloidogenesis mechanism, cross-talks of PMDs, and designing better anti-amyloid therapeutics.

## Figures and Tables

**Table 1:** List of diseases and the cross-seeding amyloid proteins found by different experimental and computational approaches.

Disease	Cross-Seeding Proteins	Ref.
Alzheimer’s disease	Aβ_42_-PrP^sc^	[57]
	Aβ_40_-Aβ_42_	[136]
	Aβ_40_-hIAPP_37_	[56]
	Aβ_24–34_-hIAPP_19–29_S20G	[137]
	Aβ-ASC specks	[138]
	Aβ-casein, fibroin, sericin, actin, and IAPP	[139]
	Tau-Aβ_42_	[53]
	Tau-αS	[55]
	Aβ_42_-Tau K18	[140]
	Aβ_42_-Tau K19	[140]
	Mutated Tau (R2)-Aβ_17–42_	[141]
	Aβ_42_-A2T Aβ_42_	[142]
	Aβ_17–42_-Tau (R2, R3 and R4)	[143]
	Tau K18-Tau K19	[144]
	Aβ_40_-hIAPP_37_	[145]
	Aβ_42_-hIAPP_37_	[146]
	Aβ_17–42_-hIAPP_37_	[147]
Parkinson’s disease	αS_human_-αS_mouse_	[148]
	αS_mouse_-N-terminal truncated αS_human_	[149]
	αS_mouse_-C-terminal truncated αS_human_	[149]
	αS_human_-Aβ	[54]
	αS_human_-quinolinic acid	[150]
	Non-β-component of αS-Aβ_42_	[151]
Prion disease	PrP_120–144 various species_-PrP_120–144 various species_	[152]
	PrP_120–144 various species_-PrP_23–144 various species_	[39]
	PrP^c^-αS	[153]
	PrP_106–126_-hIAPP	[154,155]
Type 2 diabetes	hIAPP_37_-Aβ_42_	[156]
	rIAPP_37_-hIAPP_37_	[58]
	hIAPP-Aβ	[156]
	hIAPP-rIAPP	[157,158]
	hIAPP_37_-Aβ_17–42_	[159]
